# Fundamental Mechanisms of the Cell Death Caused by Nitrosative Stress

**DOI:** 10.3389/fcell.2021.742483

**Published:** 2021-09-20

**Authors:** Fulin Wang, Qiuhuan Yuan, Fengying Chen, Jiaojiao Pang, Chang Pan, Feng Xu, Yuguo Chen

**Affiliations:** ^1^Department of Emergency Medicine, Qilu Hospital, Shandong University, Jinan, China; ^2^Chest Pain Center, Qilu Hospital, Shandong University, Jinan, China; ^3^Shandong Provincial Clinical Research Center for Emergency and Critical Care Medicine, Institute of Emergency and Critical Care Medicine of Shandong University, Qilu Hospital, Shandong University, Jinan, China; ^4^Key Laboratory of Emergency and Critical Care Medicine of Shandong Province, Key Laboratory of Cardiopulmonary-Cerebral Resuscitation Research of Shandong Province, Shandong Provincial Engineering Laboratory for Emergency and Critical Care Medicine, Qilu Hospital, Shandong University, Jinan, China; ^5^The Key Laboratory of Cardiovascular Remodeling and Function Research, Chinese Ministry of Education, Chinese Ministry of Health and Chinese Academy of Medical Sciences, The State and Shandong Province Joint Key Laboratory of Translational Cardiovascular Medicine, Qilu Hospital, Shandong University, Jinan, China; ^6^Emergency Department, The Affiliated Hospital of Inner Mongolia Medical University, Hohhot, China

**Keywords:** nitrosative stress, peroxynitrite, reactive nitrogen species, cell death, nitric oxide, cardiovascular diseases

## Abstract

Nitrosative stress, as an important oxygen metabolism disorder, has been shown to be closely associated with cardiovascular diseases, such as myocardial ischemia/reperfusion injury, aortic aneurysm, heart failure, hypertension, and atherosclerosis. Nitrosative stress refers to the joint biochemical reactions of nitric oxide (NO) and superoxide (O_2_^–^) when an oxygen metabolism disorder occurs in the body. The peroxynitrite anion (ONOO^–^) produced during this process can nitrate several biomolecules, such as proteins, lipids, and DNA, to generate 3-nitrotyrosine (3-NT), which further induces cell death. Among these, protein tyrosine nitration and polyunsaturated fatty acid nitration are the most studied types to date. Accordingly, an in-depth study of the relationship between nitrosative stress and cell death has important practical significance for revealing the pathogenesis and strategies for prevention and treatment of various diseases, particularly cardiovascular diseases. Here, we review the latest research progress on the mechanisms of nitrosative stress-mediated cell death, primarily involving several regulated cell death processes, including apoptosis, autophagy, ferroptosis, pyroptosis, NETosis, and parthanatos, highlighting nitrosative stress as a unique mechanism in cardiovascular diseases.

## Introduction

Cell death is a manifestation of the irreversible cessation and end of life, which occurs widely during development and pathological processes (Fricker et al., 2018; Green, 2019). Recent studies have shown that nitrosative stress plays an important role in the pathophysiological processes of cell death. Under physiological conditions, low-level nitrosative stress can be detected in almost all cells *in vivo*, including cardiomyocytes, endothelial cells, fibroblasts, mesenchymal stem cells, and vascular smooth muscle cells (Brunner and Wolkart, 2003; Borbely et al., 2005; Steinberg, 2013). However, little is known about nitrosative stress in pathological conditions and the degree to which nitrosative stress changes the structure and function of cells. Furthermore, the type of cell death caused by stress is also less known. Additionally, what we still have to prove is that nitrosative stress and not other stresses related to oxygen metabolism disorders affects cell death. This review focuses on the biochemistry of nitrosative stress, as well as several new forms of cell death, and highlights its role in cell death, which provides important clues for studying the mechanism of cell death-related diseases (Table 1).

**TABLE 1:** Cell death caused by nitrosative stress.

Type of cell death	When nitrosative stress increases	
	The upregulated mediators	The downregulated mediators
Apoptosis	Cytochrome *c*, Bax, caspases	Bcl-2
Autophagy	Autophagy-lysosome signaling (Beclin1, LC3, LAMP2, and cathepsin B)	Protein kinase B (PKB)–Akt pathway, and the Akt–mTOR pathway
Ferroptosis	Lethal lipid peroxidation	–
Pyroptosis	Nod-like receptor protein-3 (NLRP3) inflammasome	–
NETosis	Phosphoinositide 3-kinase (PI3K) and myeloperoxidase	Histones H2A and H2B
Parthanatos	Poly adenosine diphosphate-ribose polymerase-1 (PARP-1), and apoptosis-inducing factor (AIF)	–

### Nitrosative Stress

There are various oxygen metabolism disorders in human body, including hypoxia (West, 2017), oxidative stress (Sohal and Allen, 1990), nitrosative stress (Lim, 2019), endoplasmic reticulum stress (ERS) (Marciniak and Ron, 2006), mitochondria dysfunction (Rowlands, 2016), and carbonyl stress (Suzuki et al., 2001; Miyata and de Strihou, 2010), which are listed in Table 2. Nitrosative stress is closely associated with oxidative stress. Reactive oxygen species (ROS), such as the superoxide anion (O${}_{2}^{-}$), singlet oxygen (^1^O_2_), hydroxyl radical (OH), hydrogen peroxide (H_2_O_2_), peroxynitrite anion (ONOO^–^), and nitric oxide (NO), which are involved in oxidative stress, overlap with the formation and scavenging pathways of reactive nitrogen species (RNS) and regulate each other reciprocally (Espey et al., 2000; Maes et al., 2011). The ONOO^–^ produced can nitrate several biomolecules, including proteins, lipids, and DNA, to generate 3-nitrotyrosine (3-NT). The most important characteristic of nitrosative stress is tyrosine nitration. Protein nitration modification is a post-translational modification of proteins caused by their interaction with RNS/ROS (Ischiropoulos, 2003; Velsor et al., 2003). In recent years, various studies have shown that the formation of 3-NT is a specific biomarker of nitrosative stress. It can be used to monitor the intracellular production and localization of ONOO^–^ and the severity of cell death (Ahsan, 2013; Zhang and Wei, 2013).

**TABLE 2:** Comparison of different types of abnormal oxygen metabolism.

Type of abnormal oxygen metabolism	Explanation	Mediators
Hypoxia	Any state of insufficient physiological oxygen or insufficient tissue oxygen demand	Hypoxia-inducible factor (HIF)
Oxidative stress	The imbalance between oxidation and antioxidation and a tendency for increased oxidation	Reactive oxygen species (ROS)
Nitrosative stress	The joint biochemical reaction of nitric oxide (NO) and the free radical superoxide (O_2_^–^)	Reactive nitrogen species (RNS)
Carbonyl stress	The joint biochemical reaction of oxidation and glycosylation, increasing the accumulation of reactive carbonyl compounds from unsaturated aldehyde ketone	Advanced glycation end product (AGEs)
Endoplasmic reticulum stress	In order to cope with the accumulation of misfolded and unfolded proteins in the endoplasmic reticulum lumen and the calcium ion imbalance, cells activate signal pathways such as unfolded protein response (UPR) and endoplasmic reticulum overload response	Inositol requiring (IRE) 1α, PKR-like ER kinase (PERK), and activating transcription factor (ATF) 6α
Mitochondrial dysfunction	Mitochondria are the primary source and direct target of ROS. Abnormal respiratory chain function and ATP synthesis disorders are the main characteristics of mitochondrial dysfunction.	Adenosine-triphosphate (ATP)

#### A Brief History and the Biochemistry of Nitrosative Stress

**FIGURE 1:**
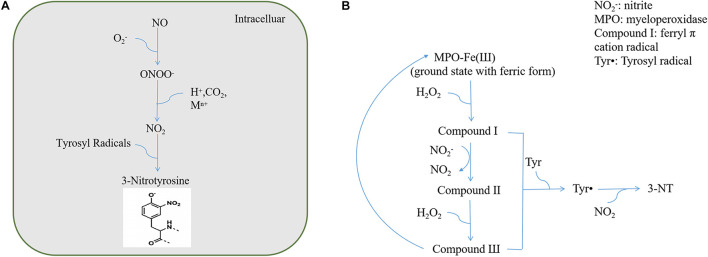
**(A)** ONOO^–^ reaction pathways. **(B)** NO_2_^–^/H_2_O_2_/heme peroxidase system.

Ohshima et al. (1990) reported the detection of 3-NT and 3-nitrophenylacetic acid in human urine and proposed that 3-NT could be used as a marker for endogenous protein nitration. In 1992, the Beckman group confirmed that ONOO^–^ can promote the nitrification of proteins such as superoxide dismutase (SOD) and CuZnSOD *in vitro* and proposed that endogenous nitrifiers can cause protein nitrification in the body (Ischiropoulos et al., 1992). Thereafter, Beckman proposed the use of the antibody method to identify nitrifying proteins and confirmed their existence in human atherosclerotic lesions and acute respiratory distress syndrome lung tissues using the immunohistochemical method (Ye et al., 1996). Subsequently, increasing studies have focused on protein tyrosine nitration from biochemical and biomedical standpoints. As extensive studies have been carried out on protein nitration in nitrosative stress injury, we primarily reviewed the biological characteristics of protein nitration. Multiple pathways can lead to protein nitration, among which ONOO^–^ and NO_2_^–^/H_2_O_2_/heme peroxidase are considered the most important pathways (Herold, 2004). The process by which NO and O_2_^–^ produce ONOO^–^ is irreversible and does not require enzyme catalysis. The protein nitration pathways induced by ONOO^–^ can be broadly divided into three categories: direct redox reactions, reactions with carbon dioxide (CO_2_), and homolysis after protonation (Alvarez and Radi, 2003; Figure 1A). Protein nitration *via* the NO_2_^–^/H_2_O_2_/heme peroxidase system also involves the formation of oxygen radicals (Bian et al., 2003; Figure 1B).

#### The Meaning of Nitrosative Stress

Nitrosative stress can promote protein tyrosine nitration, resulting in lipid peroxidation, DNA strand breaks, cell membrane damage, the inactivation of functional enzymes, and activation of cascade signal responses of cell death. Based on these basic biological functions, nitrosative stress affects diseases through various processes such as signal transduction in cells, mitochondrial energy metabolism, messenger ribonucleic acid transcription, protein post-translational modification, and ion channel function (Nobrega et al., 2019; Schiattarella et al., 2019; Paulus, 2020).

### Cell Death

Cell death is defined by the phenomenon of the irreversible cessation of life, which is one of the leading causes of treatment failure and death in multiple diseases. The process of cell death comprises many steps, and the mechanism is complicated and has become the focus of life science and medical research (Chen et al., 2020). With the deepening of research, new progress has been made on the formation and mechanisms of cell death. To date, there are at least a dozen types of cell death, including apoptosis, necroptosis, pyroptosis, oncosis, ferroptosis, entotic cell death, NETotic cell death, parthanatos, phagocytosis, lysosome-dependent cell death, autophagy-dependent cell death, alkaliptosis, and oxeiptosis (Fricker et al., 2018; Tang et al., 2019; Bedoui et al., 2020).

## Mechanisms of the Cell Death Caused by Nitrosative Stress

### Apoptosis

Nitrosative stress-mediated apoptosis is an important apoptotic pathway newly discovered following intrinsic and extrinsic apoptosis pathways associated with the pathogenesis of various diseases (Andreka et al., 2004). An increasing number of studies have shown that nitrosative stress-mediated apoptosis is involved in the pathogenesis of many diseases. To some extent, 3-NT can be used as a marker of apoptosis (Jumper et al., 2002; Zhang and Wei, 2013).

#### Mitoptosis

Studies have shown that nitrosative stress affects enzyme activity, including that of complex I (NADH dehydrogenase), complex III (cytochrome *c* reductase), complex IV (cytochrome *c* oxidase), and complex V (ATP synthase), in the mitochondrial respiratory chain and increases cytochrome *c* release from mitochondria into the cytoplasm, indicating mitochondrial dysfunction (Arnaiz et al., 1999; Mastrocola et al., 2005). Furthermore, severe nitrosative stress leads to excessive consumption of ATP, resulting in an irreversible decline in mitochondrial membrane potential (ΔΨm), which eventually leads to mitochondrial apoptosis. This indicates that nitrosative stress not only can directly cause mitochondrial damage but also can act synergistically with oxidative stress (Tao et al., 2014; Ma et al., 2018a). At present, mitochondrial permeability transition pore (mPTP) opening is considered a common pathway for endogenous apoptosis after cell injury. The generation of RNS and the opening of the mPTP are a vicious cycle of mutual promotion. Abnormal mPTP opening due to external or pathological brings about the production of large numbers of ROS, RNS, and malondialdehyde (MDA) in cells, which may lead to the conformational changes in structural proteins on the mPTP, mPTP opening, and an increase in mitochondrial permeability, which in turn leads to apoptosis (Salimi et al., 2019).

#### Endoplasmic Reticulum Stress

Nitrosative stress can synergistically induce cell apoptosis under oxidative and ERS. C/EBP-homologous protein (CHOP) and caspase-12 are key proteins involved in ERS-mediated apoptosis. Thus, nitrosative stress induces ERS-dependent apoptosis through ERS-mediated JNK activation, CHOP transcriptional activation, and caspase-12 activation (Zhao et al., 2013; Tao et al., 2014; Lv et al., 2020).

#### Caspases/Bcl-2 Family Proteins

Studies have shown that the activity of caspase-3 in nitrosative stress induced by H_2_O_2_ increases and positively correlates with the apoptosis ratio. As a direct substrate of caspase-3, Bcl-2 plays a vital role in apoptosis. Nitrosative stress causes a reduction in the expression levels of STAT3, which regulates the transcription of the Bcl-2 family of anti-apoptotic genes (Rosati et al., 2019). Additionally, nitrosative stress leads to the activation of inducible nitric oxide synthase (iNOS)/Bax/caspase-3-mediated apoptosis in the L132 cell line (Anand et al., 2014). Another study showed that α-ZAL, a natural phytoestrogen, reduces Bax expression and the expression and activity of caspase-9. These effects may be related to the inhibition of nitrosative stress (Liu et al., 2013).

#### Other Pathways in Apoptosis Signaling

Peroxisome proliferator-activated receptor gamma (PPARγ), a transcriptional regulator of energy balance, can regulate nitrosative stress and inflammation in endothelial cells. A recent study showed that salusin-β, a bioactive peptide composed of 20 amino acid residues, regulates PPARγ to attenuate nitrosative stress, thereby reducing apoptosis (Sun et al., 2017). Another protein kinase, NF-κB, stimulates IL-1β transferase protease and TNF-α gene expression and induces apoptosis. Nitrosative stress causes abnormal activation of the NF-κB signaling pathway, and its transcription activity increases, thereby triggering apoptosis signals, resulting in apoptosis (Cheng et al., 2014). Several studies found that 3-NT can bind to the C-terminus of α-tubulin through tubulin tyrosine ligase to produce tyrosine tubulin, which is insufficient to maintain microtubule stability, causing cell degeneration, eventually leading to apoptosis (Bisig et al., 2002; Peluffo et al., 2004; Blanchard-Fillion et al., 2006). Another study showed that 3-NT can be used as an indirect inhibitor of tubulin-specific carboxypeptidase, thereby blocking the production of glutamate microtubules and further causing apoptosis (Chang et al., 2002). In recent years, researchers utilize this mechanism to develop related antitumor drugs, which induce tumor cell apoptosis by mediating the nitration of tubulin in tumor cells (Xue et al., 2020).

### Autophagy

Autophagy can be divided into macroautophagy, microautophagy, and chaperone-mediated autophagy (CMA) according to the mode of transportation of degraded substances to lysosomes (Filomeni et al., 2015; Doherty and Baehrecke, 2018). In macroautophagy, intracellular autophagy-associated proteins (Atg) form autophagosomes by wrapping damaged proteins or organelles in the cytoplasm, and autophagosomes fuse with lysosomes to form autolysosomes. Autophagy lysosomes degrade the encapsulated substances, and some small molecular substances can be reused by cells. Microautophagy is a method by which lysosomes actively and directly engulf cytoplasmic components, which has rarely been explored in mammals. In CMA, some molecular chaperones, such as hsp70, can help unfolded proteins translocate into lysosomes. In recent years, with advances in research, some autophagy forms with a specific selectivity for substrate degradation, including mitochondrial autophagy (mitophagy), peroxisome autophagy (pexophagy), aggregate autophagy (aggrephagy), endoplasmic reticulum autophagy (reticulophagy), and ribosomal autophagy (ribophagy), have emerged (Kiffin et al., 2006; Coliva et al., 2019). Various studies have shown that nitrosative stress is involved in the occurrence and development of autophagy and that it can induce autophagy through multiple signal transduction pathways.

#### Macroautophagy

ROS/RNS can induce autophagy by inhibiting the protein kinase B (PKB)-Akt pathway and the Akt-mTOR pathway (Ma et al., 2018b). RNS is related to the post-translational modification of target proteins in cells, including protein thiol oxidation or S-nitrosylation related to the autophagy pathway. The accumulation of ROS/RNS generates carbonyls, whose accumulation is positively correlated with the development of autophagy (Di Meo et al., 2016; Feng et al., 2017). The autophagy-related proteins Atg3, 7, and 10 use cysteine residues to catalyze ubiquitin transfer at their catalytic sites (Ornatowski et al., 2020). The sulfhydryl groups on cysteine residues are particularly vulnerable to the modification of ROS/RNS oxidation; therefore, these proteins may be sensitive to redox signals (Filomeni et al., 2015; Varga et al., 2015). Notch 1 signaling is a key regulator of autophagy and reduces nitrosative stress. For example, genistein (Gen), a natural biologically active flavonoid found in soy, attenuates burn-induced myocardial autophagy by activating cardiac Notch 1 signaling and reducing nitrosative stress (Fang et al., 2019). In addition, nitrosative stress potentiates autophagy-lysosome signaling, which involves Beclin1, LC3, LAMP2, and cathepsin B (Han et al., 2011).

#### Mitophagy

Nitrosative stress can decrease ΔΨm and cause mitochondrial dysfunction. When ΔΨm decreases, PTEN-induced putative kinase 1 (PINK1) accumulates in the outer membrane and forms a large complex on the outer membrane surface to recruit Parkin to the damaged mitochondria, thereby causing mitochondrial autophagy (Redmann et al., 2016; Song et al., 2018). In a cerebral ischemia/reperfusion model, ONOO^–^ induced the tyrosine nitration of dynamin-related protein 1 (Drp1) peptide and recruitment of Drp1 to damage mitochondria and subsequently induce PINK1/Parkin-mediated autophagy activation, thereby promoting brain ischemia/reperfusion injury (Feng et al., 2018a,b).

#### Pexophagy

Peroxisomes are critical metabolic organelles found in nearly all eukaryotic cells and are involved in ROS/RNS metabolism. Many peroxisomes cause severe nitrosative stress during metabolism (Jo et al., 2015; Sandalio and Romero-Puertas, 2015; Olmedilla and Sandalio, 2019). A recent study showed that certain miRNAs are associated with age-related degenerative changes. MiR-142 targets endothelial PAS domain protein 1 (Epas1), which is a known pexophagy regulatory protein. MiR-142 induces RNS/ROS accumulation by inducing pexophagy (Houri et al., 2020). The accumulation of ROS/RNS inhibits mTORC1 activity in the peroxisome, leading to the active translocation of the transcription factor EB (TFEB) into the nucleus, ultimately promoting autophagic flux and specifically inducing pexophagy through ubiquitin designation (Ganguli et al., 2019; Germain and Kim, 2020).

### Ferroptosis

Ferroptosis was initially identified in small molecule erastin-induced cell death, which blocks cystine import, leading to glutathione depletion and inactivation of glutathione peroxidase 4 (GPX4) (Ursini et al., 1982; Yang et al., 2014). Ferroptosis is characterized by the accumulation of lethal lipid peroxidation, which is iron-dependent (Dixon et al., 2012; Stockwell et al., 2017; Lei et al., 2020). As the major component of lipid peroxidation, nitrosative stress was found to be related to the occurrence of ferroptosis (Uribe et al., 2018; Wu et al., 2020). Ferroptosis has been reported to be the prominent form of hepatocyte damage in Concanavalin A (ConA)-induced acute immune hepatitis, which is accompanied by RNS accumulation (Deng et al., 2020; Zeng et al., 2020). More importantly, the administration of an iNOS inhibitor or ONOO^–^ scavenger diminishes RNS levels and reduces hepatocyte ferroptosis, suggesting that RNS, downstream of Caveolin-1 (Cav-1), is an important mediator that drives the hepatocellular ferroptosis induced by ConA. In addition, nitrosative stress is regulated by indoleamine 2,3-dioxygenase 1 (IDO1) during ferroptosis. IDO1 is an important cellular heme enzyme induced by pro-inflammatory mediators in response to inflammation and contributes to restraining the T and NK cells. Upregulation of IDO1 and nitrosative stress in ConA-induced hepatic damage are suppressed by ferroptosis abolishment. DO1 deficiency leads to ferroptosis inhibition by increasing the expression of solute carrier family 7 member 11 and RNS production, and the IDO1 inhibitor reduces iNOS and 3-NT expression during ferroptosis suppression (Zeng et al., 2020). Although few studies have paid close attention to the role of nitrosative stress in ferroptosis, the nature of ferroptosis caused by lethal lipid peroxidation suggests that nitrosative stress might significantly contribute to ferroptosis in various pathological conditions.

### Pyroptosis

Nitrosative stress induces the activation of the nod-like receptor protein-3 inflammasome, which can cause pyroptosis through caspase-1- and caspase-11-mediated cytoplasmic protein gasdermin D (Mari and Colell, 2021). When acute and chronic inflammation occurs, the production rate of ONOO^–^ exceeds the ability of the endogenous ONOO^–^ defense system to scavenge it (Dedon and Tannenbaum, 2004; Galloway et al., 2014). Excessive ONOO^–^ leads to an increase in inflammatory cytokine levels and induces tyrosine nitration, ultimately exacerbating the initial damage. A previous report suggested that TNF-α can activate various transcription factors, including NF-κB, activator protein 1, and interferon regulator factor 3, which induce the expression of target genes that encode various inflammatory factors (Pei et al., 2015). Furthermore, TNF-α induces nitrosative stress by upregulating the intercellular expression of adhesion molecule-1. Adiponectin (APN), an adipocyte-derived cytokine, attenuates TNF-α-induced inflammatory response through Cav-1-mediated ceramidase recruitment and activation in an AdipoR1-dependent manner (Wang et al., 2014). In addition, the upregulation of NF-E2-related factor-2, which is mainly secreted by macrophages, reduces nitrosative stress by blocking the NF-κB signal transduction (Li et al., 2019; Nadeem et al., 2020). The inflammatory response mediates the activation of phosphoinositide 3-kinase and the AKT (PI3K/AKT) and mitogen-activated protein kinase (MAPK) signaling pathways, which may also participate in the suppression of nitrosative stress (Ma et al., 2018b; Sarkozy et al., 2018).

### NETosis

Some studies have shown that excessive intracellular RNS production in neutrophils is key to NETosis occurrence (Manda-Handzlik and Demkow, 2015; Lenin et al., 2018). Nitrosative stress mediates neutrophil activation, and activated neutrophils release neutrophil extracellular traps (NETs), a reticular structure that exerts a protective effect by surrounding and degrading pro-inflammatory cytokines (Boeltz et al., 2019). Recent research has shown that the formation of NETs induced by RNS depends on the activities of phosphoinositide 3-kinase and myeloperoxidase, as well as the selective degradation of histones H2A and H2B by neutrophil elastase (Chakraborty et al., 2011; Manda-Handzlik et al., 2020).

### Parthanatos

A recent report showed that the overactivation of poly adenosine diphosphate-ribose polymerase-1 (PARP-1) leads to cell death. It is a new form of programmed cell death, termed “parthanatos,” which differs from apoptosis and other forms of cell death (Lee et al., 2013, 2015; Tang et al., 2019). At present, research on parthanatos is in its infancy, and the molecular mechanism of its signaling pathway is still unclear. As a unique cell death pathway, parthanatos is characterized by the overactivation of PARP-1, accumulation of cytoplasmic PAR polymers, mitochondrial depolarization, and apoptosis-inducing factor (AIF) nuclear translocation. When AIF enters the nucleus, it interacts with DNA through an as-of-yet unidentified PAAN, leading to large-scale DNA fragmentation and chromatin condensation (Fatokun et al., 2014). As DNA bases are sensitive to nitrosative stress, they are often modified by ONOO^–^. One study showed that DNA nitration is upstream of PARP-1 activation. Spermidine is a naturally occurring polyamine that is widely involved in DNA replication, transcription, and translation. The use of exogenous spermidine effectively inhibits the activation of PARP-1 and DNA nitrosative stress (Kim, 2017). Under nitrosative stress, ONOO^–^ not only induces DNA single-strand breaks but also activates PARP (Buelow et al., 2009), which consumes a large amount of NAD^+^, resulting in energy depletion and necrotic cell death. One study showed that DNA nitration is upstream of PARP-1 activation (Barany et al., 2017; Horvath et al., 2018). The initial signaling of PARP-1 in mitochondria is mediated by the downstream activation of JNK-1, a stress-activated protein kinase (SAPK). When nitrosative stress occurs, JNK-1 is activated and participates in the regulation of parthanatos by regulating the intracellular ROS/RNS levels (Zheng et al., 2017).

## Nitrosative Stress and Cardiovascular Diseases

Cardiovascular diseases have become the most important causes of death worldwide. Therefore, clarifying its pathophysiological mechanisms is crucial for formulating prevention and treatment strategies. From a cardiovascular aspect, nitrosative stress is associated with the pathophysiological processes. Nitrosative stress can lead to key protein tyrosine nitration related to contractility, metabolism, and antioxidant defense mechanisms of the myocardium and skeletal muscle (Breitkreuz and Hamdani, 2015; Mozos and Luca, 2017). Furthermore, it induces myocardial hypertrophy, fibrosis, or cell death by activating inflammatory responses and stress signals (such as apoptosis and autophagy) (Frati et al., 2017). Myocardial ischemia/reperfusion injury and heart failure are the two main pathophysiological alterations associated with nitrosative stress. Recent studies demonstrated that melatonin receptor 2 (MT2) protects cardiomyocytes from nitrosative stress injury through the MT2/Notch1/Hes1/RORIα signaling pathway in a mouse myocardial ischemia/reperfusion model. Moreover, TNF-α-converting enzyme (TACE) protects against MRI *via* Notch1-mediated suppression of nitrosative stress. These results demonstrate that the upregulation of Notch1 may alleviate nitrosative stress-mediated myocardial ischemia/reperfusion injury (Pei et al., 2015; Yu et al., 2018; Zhang et al., 2020). In addition, ONOO^–^ can interfere with the cellular calcium transport system, such as the oxidation of sulfhydryl groups of Na^+^/Ca^2+^ exchangers, resulting in Na^+^/Ca^2+^ exchanger protein dysfunction (Kitao et al., 2010). In heart failure, nitrosative stress causes sarcoplasmic reticulum Ca^2+^-ATPase tyrosine nitration (Schoneich et al., 1999; Lokuta et al., 2005), further aggravating the dysfunction of left ventricular and vascular systolic functions (Kennedy et al., 2015; Schiattarella et al., 2019). Heart failure induces excessive production of iNOS by macrophages and cardiomyocytes, leading to the loss of myocardial contractility and decreased reactivity of β-adrenergic receptors (Kingery et al., 2017). Nitrosative stress adversely affects the regulation of muscle fiber energy. Myofibril creatine kinase plays an essential role in regulating cardiomyocyte contraction in patients with heart failure and is more susceptible to nitration modification. Creatine kinase activity can be inhibited by nitrosative stress, resulting in the decline of myocardial contractile function and promotion of the occurrence and development of heart failure (Mihm et al., 2001a,b).

## Conclusion

Nitrosative stress falls under oxidative stress in the traditional category. However, in recent years, a growing body of knowledge has revealed that nitrosative stress, as an independent special biochemical phenomenon in the process of cell death, has unique pathophysiological characteristics that differ from those of oxidative stress in a general sense. This is because of the high responsiveness and short half-life of RNS. However, current research methods have not been able to accurately identify the RNS that induces nitrosative stress. In addition, there is a lack of practical methodologies for the positioning and quantification of nitrosative stress. Although a few antibodies can detect 3-NT, protein tyrosine nitration does not fully reflect the level of nitrosative stress. Therefore, future breakthroughs in biological methodology for detecting nitrosative stress will promote the development of research in this field and deepen our understanding of nitrosative stress biology. During nitrosative stress, the specific target protein tyrosine nitration in different diseases differs. Further studies on the relationship between specific protein tyrosine nitration and diseases will help reveal the pathogenesis of related diseases and identify new targets for drugs. The mechanisms underlying cell death due to nitrosative stress include target protein tyrosine nitration, mitochondrial dysfunction, and cell membrane destruction, which eventually lead to cell death. Revealing the mechanism of cell death induced by nitrosative stress is of great significance for the development of drugs targeting nitrosative stress. In recent years, significant progress has been made in determining the role of nitrosative stress injury in cell death; however, some key problems, such as the concentration threshold of ONOO^–^ damage to various important cellular organs and the corresponding pathophysiological effects, have not been addressed. In summary, research on the mechanism of cell death induced by nitrosative stress helps us to further reveal the pathogenesis of cell injury, explore new sub-therapeutic targets, and provide new ways to prevent and treat diseases related to cell death. Cardiovascular disease is the first enemy of human health. Understanding nitrosative stress will help bridge the gap in the relationship between cell death and cardiovascular disease, thereby providing vital information for improving human health.

## Author Contributions

YC, FX, and FC designed the framework. FW and QY wrote the manuscript. JP and CP revised the manuscript. All authors read and approved the final version of the manuscript for publication.

## Conflict of Interest

The authors declare that the research was conducted in the absence of any commercial or financial relationships that could be construed as a potential conflict of interest.

## Publisher’s Note

All claims expressed in this article are solely those of the authors and do not necessarily represent those of their affiliated organizations, or those of the publisher, the editors and the reviewers. Any product that may be evaluated in this article, or claim that may be made by its manufacturer, is not guaranteed or endorsed by the publisher.
